# Benefits of Hesperidin for Cutaneous Functions

**DOI:** 10.1155/2019/2676307

**Published:** 2019-04-02

**Authors:** Mao-Qiang Man, Bin Yang, Peter M. Elias

**Affiliations:** ^1^Dermatology Hospital, Southern Medical University, Guangzhou 510091, China; ^2^Department of Dermatology, University of California San Francisco and Veterans Affairs Medical Center, San Francisco, CA 94121, USA

## Abstract

Hesperidin is a bioflavonoid, with high concentration in citrus fruits. In addition to its well-known benefits for cardiovascular function, type II diabetes, and anti-inflammation, recent studies have demonstrated multiple benefits of hesperidin for cutaneous functions, including wound healing, UV protection, anti-inflammation, antimicrobial, antiskin cancer, and skin lightening. In addition, hesperidin enhances epidermal permeability barrier homeostasis in both normal young and aged skin. The mechanisms by which hesperidin benefits cutaneous functions are attributable to its antioxidant properties, inhibition of MAPK-dependent signaling pathways, and stimulation of epidermal proliferation, differentiation, and lipid production. Because of its low cost, wide availability, and superior safety, hesperidin could prove useful for the management of a variety of cutaneous conditions.

## 1. Introduction

In humans, no organ has attracted as much attention as the skin does, because of both cosmetic and medical concerns. For cosmetic concerns, average daily costs of facial care for an American woman can be as much as $8.00 [1]. In 2017, the sale value of skin care products exceeds $26 billion per year in China alone [2]. Recent studies showed that topical applications of certain skin care products exert a variety of benefits for both chronic and photoaged skin, antimicrobials, and anti-inflammation [3–7]. Because use of skin care products has become increasingly popular, much work has been focused on the identification of ingredients with multiple benefits on the skin in the development of skin care products.

Because skin suffers from as many diseases as any other organ in the body, proper management of cutaneous conditions is of substantial importance. Over a lifetime, everyone will eventually suffer from some cutaneous problems, because the skin interfaces with the environment, making it more vulnerable to external physical, chemical, and microbial stress. In addition to their psychosocial impact and the quality of life for affected patients and their families, certain chronic cutaneous disorders can also contribute to the development of other systemic diseases. For example, both psoriasis and eczematous dermatitis increase circulating levels of proinflammatory cytokines [8–10], which appear to play a pathogenic role in the development of cardiovascular diseases, obesity, type II diabetes, and Alzheimer's disease [11–14]. Because of its vast size, even subclinical inflammation in the skin can dramatically increase serum cytokine levels, which could be linked to some of these age-associated disorders [12, 15]. Due to the complexity of cutaneous functions and the potential risk of developing multiple disorders in the skin, ingredients that exert multiple benefits to the skin are much desirable. In search for these ingredients, hesperidin would appear to be a potential candidate. Studies have demonstrated that both topical and systemic administrations of hesperidin can benefit a variety of cutaneous functions in both normal and diseased skin. In this review, we comprehensively summarize the benefits of hesperidin for cutaneous functions.

## 2. Sources and Chemical Properties of Hesperidin

Hesperidin was first isolated from the inner portion of orange peels in 1828. Hesperidin together with other similar bioflavonoids was formerly called “vitamin P” (reviewed in [16]). Hesperidin is abundant in citrus fruits, including lemon, orange, lime, and grapefruit. The content of hesperidin in citrus fruits varies greatly with species, part of the fruit itself, geographic sites of cultivation, and processing procedures (Table 1) [17–22]. For example, hesperidin content in fresh Satsuma pulp is 73 mg per kilogram and 157 mg per kilogram in fresh peel [20]. Generally, hesperidin content is higher in citrus peel than in the other parts of the citrus fruits. But lemon seeds contain more hesperidin than peel by methanol extraction [23]. Hand-squeezed Florida orange juice contains 335–351mg hesperidin per litter while Israel Ortanique citrus juice contains 273–287 mg per litter [24]. Juice from pigmented citrus contains more hesperidin than that from nonpigmented citrus [25]. It is likely that immature citrus may contain more hesperidin than ripen citrus does [26]. Pasteurization with heat did not decrease hesperidin content in citrus juice at least stored at 4°C for up to 12 days. Instead, hesperidin content increases following pasteurization of citrus juice at 90°C for 20 seconds [27]. Hesperidin content ranges from 555 to 761 mg per litter in single-strength juice and from 470 to 614 mg per litter in concentrated juice, suggesting that processing procedure affects hesperidin content in citrus juice [28]. In addition to citrus fruits, peppermint (Mentha x piperita L.) also contains hesperidin, whose content increases following UVB irradiation [29]. Methanol extract of Porphyra dentata, a red edible seaweed, contains 5% hesperidin [30].

Hesperidin (3,5,7 trihydroxyflavanone 7-rhamnoglucoside, C_28_H_34_O_15_) is also named hesperetin 7-rutinoside or 7-O-glycoside hesperitin, with a molecular weight of 610.57. The melting and boiling points of hesperidin are 250-255°C and 576.16°C, respectively. It is stable for at least for 2 years if stored at -20°C. Although hesperidin alone barely dissolves in aqueous solution, it dissolves well in both propylene glycol and poly(ethylene glycol)-400 [71]. Reaction of hesperidin with chitooligosaccharide yields hesperidin-chitooligosaccharide complex, which renders it water soluble and further exhibits superior antioxidant activity to hesperidin alone [72]. Moreover, mix of hesperidin and other flavonoids such as theaflavin-3 3′-digallate can increase the solubility of hesperidin in 10% dimethyl sulfoxide [73]. Additionally, alpha glucosyl hesperidin is also water soluble and therefore commonly used in both topical and systemic preparations. Pertinently, the area under the curve for serum hesperidin in rats was over 3-fold higher following orally administrations of glucosyl hesperidin than hesperidin itself [74].

## 3. Safety

Hesperidin is generally safe for both topical and systemic administrations. Topical applications of 2% hesperidin for 9 days caused no adverse cutaneous reactions in mice [32]. Similarly, intragastrically given Daflon-500 mg, containing 10% hesperidin, at daily dose of 100 mg did not show signs or symptoms of side effects in rats [75]. Likewise, oral administrations of diets, containing either methyl hesperidin or hesperidin, did not show signs or symptoms of side effects in mice and rats, either [76, 77]. Moreover, no adverse events were observed in mice followed daily intraperitoneal injections of phosphorylated hesperidin at dose of 20mg/kg body weight for over 4 weeks [78]. Although one study showed that orally given Daflon-500 mg twice-daily for 60 days caused minor, temporal side effects such as headache and faintness [79], other studies showed that oral Daflon-500 mg is safe in humans [80, 81].

## 4. Benefits of Hesperidin for Cutaneous Functions

Nowadays, the benefits of bioflavonoids, including hesperidin, on human health have been well appreciated. A large number of studies have demonstrated that systemic administrations of hesperidin exhibit benefits for a variety of diseases, including cardiovascular diseases, diabetes, Alzheimer's disease, and cancer [82–88]. Likewise, the benefits of hesperidin on various cutaneous conditions have also been well illustrated (Table 2).

### 4.1. Epidermal Permeability Barrier Function

Epidermal permeability barrier, residing in the stratum corneum, prevents movement of agents and water through the stratum corneum. Importantly, recent studies demonstrate that epidermal permeability barrier plays crucial role in the pathogenesis of both cutaneous and possibly systemic disorders [89–91]. Thus, skin care product makers have been striving to develop products that can potently improve epidermal permeability barrier function. Because of the high incidence of adverse cutaneous reactions to skin care products, identification of safe and effective ingredients is becoming emergent [92, 93]. Our group has demonstrated that twice-daily applications of 2% hesperidin to young mouse skin for 6 days accelerated permeability barrier recovery in a model of acute barrier disruption although basal transepidermal water loss rates, stratum corneum hydration, and skin surface pH remained unchanged [31]. Aged skin displays delayed permeability barrier recovery and elevated skin surface pH [94, 95], which both possibly contribute to the development of certain aging-associated disorders. Regimens that can improve epidermal permeability barrier function, particularly at gene levels, are limited. At least on study showed that topical applications of 2% hesperidin twice-daily for 9 days markedly accelerated permeability barrier recovery, along with significant reduction in skin surface pH in aged mice [32]. In addition to normal skin, topical hesperidin can also prevent abnormalities of epidermal permeability barrier induced by topical glucocorticoid in mice. For example, repeated topical applications of glucocorticoids delayed barrier recovery. But if hesperidin was topically given following each glucocorticoid application to mice, abnormalities in both permeability barrier recovery and skin surface pH were normalized [33]. No adverse reactions were observed following topical applications of hesperidin. Taken together, these studies demonstrate that topical hesperidin improves epidermal permeability barrier function in both normal and glucocorticoid-disrupted skin.

### 4.2. UV-Induced Cutaneous Damage

Because the skin is the outermost layer of the body, it is more vulnerable to ultraviolet (UV) irradiation, leading to the development of photoaging and other cutaneous disorders such as actinic keratosis and skin cancers [96, 97]. Thus, protection against UV irradiation can prevent and/or mitigate UV-induced cutaneous damage. Study showed that treatment of keratinocytes with 50 *μ*M hesperidin could cause over 70% reduction in apoptotic index induced by UVB irradiation in comparison to vehicle-treated keratinocytes [34]. In addition to UVB, hesperidin can also protect keratinocytes from UVA-induced damage. Li et al. [35] reported that treatment of keratinocytes with hesperidin for 24 hr induced a dose-dependent increase in viability of UVA-irradiated keratinocytes. Pretreatment of keratinocytes with hesperidin at a dose of 220 *μ*g/ml also significantly reduced UVA-induced oxidative stress and expression levels of proinflammatory cytokines. These data indicate that hesperidin protects keratinocytes from both UVA- and UVB-induced damage.

The wavelength of UVA is 320-400 nm, which can penetrate into the dermis, leading to premature skin aging (photoaging) upon repeated exposure. It appears that hesperidin can also attenuate UVA-induced damage to fibroblasts. Because hesperidin is hydrolyzed to hesperetin by the gut microbiota and absorbed by passive transport in the large intestine [36], some studies use hesperetin instead of hesperidin. Bae et al. [37] reported that treatment of UVA-irradiated human fibroblasts with 0.1% Citrus unshiu peel extract, containing hesperetin (metabolite of hesperidin), decreased expression levels of *β*‐galactosidase, matrix metalloproteinase-1, and the number of senescent cells. Moreover, pretreatment of human fibroblasts with hesperetin glucuronides induced a 25% protection against UV‐A‐induced necrotic cell death [38].

Hesperidin not only protects cells against UV-induced damage* in vitro*, but also protects the skin from UV-induced damage* in vivo*. Pretreatment of mouse skin with topical hesperidin could prevent UVB-induced elevations in cutaneous cytokine expression and lipid peroxidation, while increasing expression levels of antioxidant enzymes such as glutathione peroxidase-1, glutathione reductase, and heme oxygenase-1 in mouse skin, following either single or multiple UVB irradiation [39, 40]. In addition, topical applications of hesperidin also markedly prevented UVB irradiation-induced erythema, edema, and epidermal proliferation [39]. Moreover, intraperitoneal administrations of hesperidin methyl chalcone can prevent UVB irradiation-induced reductions in antioxidant capacity and elevations in both cutaneous cytokine expression and myeloperoxidase activity [41]. Lee et al. [42] reported that daily drinking water containing hesperidin attenuated a number cutaneous abnormalities induced by repeated UVB irradiation, including compromised epidermal permeability barrier, promoted wrinkle formation, increased cytokine expression, and both expression levels and activity of matrix metalloproteinase-9. Furthermore, pretreatment of mice with topical hesperidin could enhance repair of DNA damage induced by UVB irradiation [43]. Finally, topical hesperetin lowered transepidermal water loss by ≈50% in guinea pigs subjected to repeated UVB irradiation [44]. Collectively, either topical or oral administrations of hesperidin can protect skin from damage induced by both UVA and UVB irradiation.

### 4.3. Melanogenesis

For beauty concern, skin whitening is very popular, particularly in Asia. Hesperidin has long been used as a skin whitening agent although the results of its effects on melanogenesis are controversy. Study showed that treatment of murine B16-F10 melanoma cells with 20 *μ*g/mL citrus extract (containing 362.3 ± 16.7 *μ*g/mL hesperetin) induced onefold increase in melanin content, while hesperidin alone also increased melanin content by over 20% [45]. Likewise, 50*μ*M hesperetin increased melanin content by over 80% in murine B16-F10 melanoma cells [46]. In contrast, other studies demonstrated that hesperidin did not affect melanin production in B16F10 murine melanoma cells [47–49]. However, most of other studies showed that both citrus extract and hesperidin inhibited melanogenesis in both murine B16-F10 melanoma cells and human melanocytes [36, 44, 50–53]. For example, treatments with 50*μ*M hesperidin for 48-72 hr induced 60% reduction in melanin content in murine B16-F10 melanoma cells and ≈30% reduction in human melanocytes [50]. Topical applications of 0.2% hesperidin to reconstructed human epidermis for 14 days reduced pigment by ≈25% [49]. In addition, topical applications of hesperetin, a metabolite of hesperidin, lightened skin in UVB-induced hyperpigmentation [44]. Thus, topical applications of hesperidin and its metabolite can reduce epidermal pigmentation in both normal and UVB challenged skin.

### 4.4. Cutaneous Wound Healing

Cutaneous wounds are very common while management of cutaneous wounds is still a challenge, particularly in certain conditions such as diabetic and venous wounds. A number of studies have demonstrated that hesperidin accelerated wound healing both in* vitro* and in* vivo*. Wessels et al. [54] reported that addition of a culture medium containing 0.05 % hesperidin for 24 hr accelerated wound closure by 39% in comparison to vehicle control in* in vitro* scratch models. In diabetic rats, wound almost completely healed (97%) following orally given hesperidin (100 mg/kg body weight) for 21 days while the wound did not close at all in the vehicle-treated controls [55]. In diabetic rats, the benefits of oral hesperidin on wound healing and other biomarkers, including serum glucose and glycated hemoglobin, were comparable to insulin treatments [56]. Besides diabetic wound, treatment of wound in venous insufficient subjects is also troublesome. Although wound healing times were similar in patients treated with oral diosmin/hesperidin (450/50 mg) and with pycnogenol [57], more patients were completely healed in diosmin/hesperidin-treated group than in placebo controls (32% versus 13%) after 2-month treatment [58]. Moreover, either topical or oral administrations of hesperidin shortened wound healing time ≈3 days in *γ* ray-irradiated mice [59, 60]. These data demonstrate that either topically or orally given hesperidin can accelerate cutaneous healing under various conditions.

### 4.5. Inflammation

Benefits of flavonoids on both systemic and local inflammation have been demonstrated [98, 99]. Because nitric oxide is an inflammatory mediator, it is often used as a biomarker to evaluate inflammatory response. In* vitro* study demonstrated that treatment of mouse RAW 264.7 cells with lipopolysaccharide (LPS) for 24 hr induced over 9-fold increase in nitrite levels. But addition of hesperidin (250 *μ*g/ml) to culture medium lowered nitrite levels by ≈75% in LPS-treated cells [30]. Yang et al. [61] performed a similar study using hesperetin and its metabolites. The results showed that stimulation of RAW 264.7 cells with LPS markedly increased in both nitric oxide and inducible nitric oxide synthase mRNA levels, which both significantly decreased by coincubation of RAW 264.7 cells with LPS and 10 *μ*M hesperetin metabolite. Interestingly, hesperetin only at lower dose (1*μ*M) lowered nitric oxide and inducible nitric oxide synthase mRNA levels, but hesperetin at dose of 10 *μ*M had no effect.

The skin serves as the first line of defense against to external stimuli. Keratinocytes can produce and release proinflammatory cytokines upon stimulation [90]. Hesperidin can lower cytokine production in keratinocyte cultures. For instance, prior to challenge with H_2_O_2_ (100 *μ*M), treatment of keratinocytes with hesperidin (20 *μ*g/ml) for 2 hr could inhibit IL-8 and TNF*α* production by 96% and 78%, respectively [62]. Expression levels of cyclooxygenase-2 (COX-2) protein and mRNA also significantly decreased in keratinocytes cotreated with hesperidin versus treated with alone H_2_O_2_. Evidence indicates that hesperidin can inhibit bacterial pathogen-induced cytokine production, too. Incubation of keratinocytes with both Propionibacterium acnes and 5-5 *μ*g/mL of hesperidin for 24 hr inhibited IL-8 and TNF*α* production by 49% and 71%, respectively, which were comparable to the levels inhibited by dexamethasone treatment [63]. Moreover, pretreatment of hesperidin methyl chalcone (0.2 mg/ml) also dramatically decreased the proportion of dilated vessels (48% inhibition), total vessel area (72% inhibition), and IL-8 production (79% inhibition) in human skin explants following stimulation with substance P [64]. Thirty minutes prior to subcutaneous injection of carrageenan (1%), subcutaneous injection of hesperidin at doses of 50 and 100 mg/kg reduced the paw edema by 47 and 63%, respectively, within 5 hr [65]. Hesperidin at dose of 100 mg/kg also decreased dextran-induced edema by 33%. The efficacy of hesperidin on edema was comparable to that produced by oral indomethacin (10 mg/kg). However, hesperidin did not prevent histamine-induced paw edema. Pelzer et al. [66] reported that intraperitoneal injection of hesperidin could also inhibit carrageenan-induced paw edema by 36 to 40% within 7 hr. One hour prior to edema induction with carrageenan on the paw, oral administration of hesperidin (40 mg/kg/ body weight) could decrease the edema by 50%, 51%, 63% and 77 %, respectively, while indomethacin (10mg/kg) decreased the edema by 65%, 71%, 72% and 74%, respectively, after 1, 2, 3, and 4 hours [67]. These results indicate that hesperidin can prevent and treat cutaneous inflammation induced by various agents.

### 4.6. Cutaneous Cancers

In addition to the preventive and therapeutic benefits for other cancers [100, 101], studies showed that hesperidin and its metabolite also benefit cutaneous cancers. Smina et al. [68] showed that treatment of A431 cells with hesperetin at as low as 10 *μ*M induced DNA fragmentation along with significant increase in Bax, an apoptotic protein, expression while reducing expression levels of cyclin B1, D1, D3, and E1 proteins by over 1-fold. A similar study also demonstrated that incubation of A431 cells with 10 *μ*M hesperidin induced over 10-fold increase in apoptosis and DNA damage [69]. In* vivo* study demonstrated that hesperidin can prevent the development of skin tumor. For example, daily subcutaneous injection of 125 *μ*l of 1% hesperidin 1 week prior to induction of skin tumor by topical 12-O-Tetradecanoylphorbol-13-acetate (TPA) resulted in reductions in tumor incidence by 50% and the number of papillomas per mouse by 48% after 20 weeks of TPA applications [70]. Thus, hesperidin could be an alternative regimen for preventing and treating cutaneous cancers.

### 4.7. Other Cutaneous Functions

Evidence also indicates benefits of hesperidin on other cutaneous functions. Orally given hesperidin 30 min prior to irradiation with *γ* ray upregulated expression levels of mRNA for vascular endothelial growth factor by over 25 folds [102]. Hesperidin exhibited antimicrobial activity, including the common pathogens in the cutaneous infections such as* Staphylococcus aureus, Candida albicans, Candida tropicalis,* and* Streptococcus pyogenes*, with the minimum inhibitory concentration of 8.25% for both* Candida albicans* and* Staphylococcus aureus* [103–106]. A clinical trial on humans showed that orally given hesperidin (500 mg/daily) for 28 days markedly reduced facial roughness and 33% reduction in beta-galactosidase, a biomarker of aging, by 6 months [107].

## 5. Mechanisms

Although a line of evidence shows that hesperidin benefits a number of cutaneous functions, the underlying mechanisms by which hesperidin acts are unclear yet. It appears that hesperidin and its metabolite act via a variety of mechanisms depending on which function regulated by hesperidin.

### 5.1. Improvements in Epidermal Permeability Barrier Function

Formation of epidermal permeability barrier is highly regulated by multiple keratinocyte functions, including proliferation, differentiation, lipid production, acidification, and antimicrobial peptide expression [108]. In young mice, topical hesperidin mainly upregulated expression levels of filaggrin and stimulated keratinocyte proliferation [31] while in aged mice, topical hesperidin upregulated expression levels of a whole panel of mRNA associated with epidermal permeability barrier, including sodium/hydrogen exchanger (NHE1), secretory phospholipase A2 (sPLA2), differentiation-related proteins (filaggrin, involucrin, and loricrin), lipid synthetic enzymes (fatty acid synthase; 3-hydroxy-3-methyl-glutaryl-coenzyme A reductase), and lipid transport protein (ATP-binding cassette subfamily A member 12) [32]. However, in glucocorticoid-treated mouse epidermis, topical hesperidin dramatically increased filaggrin protein and glutathione reductase mRNA expression, *β*-glucocerebrosidase activity, and epidermal proliferation [33]. Thus, the underlying mechanisms by which topical hesperidin improves epidermal permeability barrier function could be attributable to upregulation of these functions, depending on the skin conditions.

### 5.2. Protection against UV Irradiation

UV irradiation causes skin damage mainly in three aspects, i.e., oxidative stress, DNA fragmentation, and inflammation. Nuclear factor erythroid 2-related factor 2 (Nrf2) is a mast regulator of antioxidant system in the cells [109, 110]. Nrf2 deficiency accelerated UV irradiation-induced photoaging and inflammation [111, 112] while activation Nrf2 can protect UV irradiation-induced apoptosis and inflammation [113, 114]. In addition to upregulation of Nrf2 expression in the senescent rat heart [115], methylhesperidin, methylated derivative of hesperidin, enhanced translocation of Nrf2 from cytoplasm to nuclear, resulting in upregulation of antioxidant-related gene expression and reduction in reactive oxygen species, consequently leading to protection of epidermal keratinocytes against UVB-induced damage in keratinocyte cultures [116]. Hesperidin-induced reductions in DNA damage and cytokine expression appear to be due to decreased oxidative stress in UV-irradiated keratinocytes [34, 35]. Moreover, hesperidin inhibited UVB irradiation-induced increase in expression levels of phosphorylation of mitogen-activated protein kinase (MAPK) and extracellular signal-regulated kinases (ERK) in mice [42]. Hence, UV protection of hesperidin can be primarily due to upregulation of antioxidant and downregulation of MAPK/ERK signaling pathway.

### 5.3. Melanogenesis

Skin pigmentation is determined by both melanogenesis and melanosome transport. Proteins involved in melanogenesis include tyrosinase, tyrosinase-related proteins (TRP) and microphthalmia-associated transcription factor (MITF). Upregulation of expression levels of tyrosinase, TRPs, and MITF can increase melanin production [117, 118]. A number of studies demonstrated that hesperidin decreased expression levels and activity of tyrosinase, TRPs, and MITF in both B16 mouse melanoma cells and human melanocytes [36, 50–52]. Moreover, hesperidin could activate *α* adrenergic receptor, leading to induction of aggregation of melanophores in B. melanostictus, suggesting that hesperidin-induced skin lightening is mediated by adrenergic receptor [119]. Another mechanism whereby hesperidin lightens skin could be attributable to inhibition of melanosome transport in melanocytes, instead of inhibition of melanogenesis [49]. Therefore, hesperidin lightens skin via inhibition of both melanogenesis and melanosome transport.

### 5.4. Acceleration of Cutaneous Wound Healing

Wound healing is involved in cell proliferation and migration and vascular formation. Activation of tumor growth factor beta (TGF-*β*) signaling and vascular endothelial growth factor (VEGF) expression are crucial for wound healing and restoration of epidermal permeability barrier function [120–123]. Study showed that oral administration of hesperidin (50 mg/kg body weight) increased TGF-*β* and VEGF-c mRNA expression by over 2-fold in a diabetic model of Sprague Dawley rats [55]. Additionally, expression levels of mRNA for VEGF receptors also increased following oral administrations of hesperidin at a dose of 50 mg/kg body weight [56]. Oxidative stress can impede wound healing in both diabetic and normal conditions while antioxidants can improve wound healing [124–126]. In diabetic rats, orally given hesperidin significantly increased cutaneous SOD and GSH content while reducing MDA content, along with acceleration of cutaneous wound healing, indicating the antioxidant property of hesperidin contributes to its acceleration of cutaneous healing [55, 56].

Inflammatory response is required for wound healing in early phase. Topical applications of cytokines such as recombinant human granulocyte-macrophage colony-stimulating factor accelerated cutaneous wound healing [127–129]. However, excessive inflammation can delay wound healing and potentially cause scar formation [130]. Accordingly, anti-inflammation could accelerate cutaneous wound healing [131, 132]. Hesperidin decreased cytokine expression, including TNF*α*, IL-6, and IL-8, in both rat skin and human keratinocyte cultures [56, 62, 63]. Taken together, hesperidin-induced acceleration of cutaneous wound healing can be attributable to upregulating expression of VEGF, antioxidant enzymes, and anti-inflammation.

### 5.5. Attenuation of Inflammation

Development of inflammation is a complex process involving interactions of a number of molecules in various signaling pathways, including p38 mitogen-activated protein kinase (MAPK) pathway [133]. Inhibition of p38 MAPK signaling pathway can markedly lower expression of IL-1*β* and IL-6, IL-8, IL-18, and TNF*α*, in both macrophage culture and mice [134, 135]. Study showed that, prior to H_2_O_2_ stimulation, treatment of keratinocytes with hesperidin for 2 hr induced over 50% reduction in NF‐*κ*B and phosphorylated p38 MAPK in comparison with those without pretreatment with hesperidin [62]. Likewise, treatment of mouse RAW 264.7 cells with hesperetin metabolite almost completely reversed lipopolysaccharide-induced increase in NF‐*κ*B expression in addition to reductions in phosphorylated p38 MAPK and c-Jun N-terminal kinase 1/2 [61]. Thus, hesperidin-induced inhibition of p38 MAPK signaling pathway could contribute its attenuation of inflammation.

### 5.6. Treatment of Cutaneous Cancers

Although studies have demonstrated that hesperidin and its metabolite exerted anticancer property both in* vitro *an in* vivo* [68–70], the underlying mechanisms are inconclusive. Zhao et al. [69] showed that treatment of A431 human skin carcinoma cells with hesperidin (25 *μ*M) for 72 hr induced over 1-fold increase in reactive oxygen species, 40% reduction in intercellular ATP content and 80% reduction in SOD content. Using other cell lines, it has been demonstrated hesperidin can induce endoplasmic reticulum stress and activate caspase-9, caspase-8, and caspase-3 activities [136–138]. Moreover, TGF*β*-Smad signaling pathway, particularly Smad3, plays a key role in the development of certain cancers [139, 140]. Previous study revealed that oral administrations of hesperidin (100 mg/kg body weight) for 18 weeks inhibited TGF*β*-Smad3 signaling and prevented development of hepatic cancer [141]. Furthermore, several studies have demonstrated that reduction in the formation of micronucleus could contribute to the anticancer properties of hesperidin, at least, in models of some chemically induced cancers. Hesperidin-induced protection in *γ* ray-induced DNA damage could also be attributable to the reduction in the formation of micronucleus. Additionally, hesperidin can increase apoptosis of cancer cells via upregulation of peroxisome proliferator-activated receptor *γ* expression.

Signaling pathways involved in the action of anticancers induced by hesperidin include (a) inhibition of Janus kinase/signal transducer and activator of transcription (JAK/STAT) pathway, activation of which can enhance proliferation, invasion, and metastasis of cancer cells; (b) inhibition of phosphatidylinositol-3-kinase (PI3K)/Akt and the mammalian target of rapamycin (mTOR) pathways, which also play crucial role in proliferation, survivability, invasion and metastasis of cancer cells upon activation; (c) activation of Notch pathway, in which activation of Notch receptors leads to translocation of Notch into nucleus and binding to target genes, resulting in increased apoptosis [142]. Of course, other signaling pathways such as MAPK-ERK, Wingless, and INT-1, NF-*κ*B, and cyclooxygenase-2 pathways have also been proposed to be involved in hesperidin-induced prevention and inhibition of cancers [142]. Therefore, anticancer benefit of hesperidin is likely via multiple mechanisms, including inhibition of TGF*β*-Smad3, PI3K/Akt, and JAK/STAT signaling pathways, activation of Notch pathway, reduction in ATP content, and induction of apoptosis.

### 5.7. Antioxidation

Oxidative stress has been linked to the development of a variety of disorders [143, 144]. As mentioned above, Nrf2 is a key regulator of antioxidant system [109, 110]. In normal condition, Nrf2 is present as Nrf2/Keap1 complex in the cytoplasm and degraded in proteasome. Upon oxidative stress, Nrf2 is separated from Keap1 and enters into nucleus, where Nrf2 can bind to antioxidant response element (ARE) within gene promoter region, leading activation of gene transcription, including reactive oxygen scavenging enzymes such as superoxide dismutase, glutathione peroxidase, glutathione reductase, catalase, and heme oxygenase 1 [109, 145], which all play crucial role in protecting cells from oxidative stress. Previous studies have shown that hesperidin and its metabolite, hesperitin, can increased Nrf2 expression while stimulating degradation of Keap1, resulting in an increase in nuclear translocation of Nrf2 and production of antioxidant enzymes along with reduction in oxidation [109, 146–148]. Thus, antioxidant property of hesperidin also largely accounts for its benefits in the skin.

In conclusions, either topical or systemic administrations of hesperidin appear to benefit multiple cutaneous functions via divergent mechanisms. Taking citrus juice or other hesperidin-containing products likely could benefit cutaneous functions. However, proper clinical trials are required to validate the benefits of hesperidin for various cutaneous conditions.

## Figures and Tables

**Table 1:** Hesperidin content in citrus fruits.

Species	Geographic Site of Cultivation	Extraction Solvent	Peel (mg/g dried)	Ref.
C. reticulate “Erythrosa”	Shimen County, Hunan, China	Methanol	74.236 ± 0.845	[17]
C. reticulate “Unshiu”	Chahe, Hubei, China	Methanol	60.540 ± 0.763	[17]
C. reticulate “Unshiu”	Yangshuo County, Guangxi, China	Methanol	70.232 ± 0.487	[17]
C. reticulate “Subcompressa”	Yongquan, Zhejiang, China	Methanol	100.525 ± 1.398	[17]
C. reticulate “Subcompressa”	Huangyan, Zhejiang, China	Methanol	62.678 ± 0.697	[17]
C. reticulate “Chachi”	Gujin, Jiangmen, Guangdong, China	Methanol	62.919 ± 0.543	[17]
C. reticulate “Chachi”	Huicheng, Jiangmen, Guangdong, China	Methanol	59.012 ± 0.787	[17]
C. reticulate “Chachi”	Luokeng, Jiangmen, Guangdong, China	Methanol	74.973 ± 0.845	[17]
C. reticulate “Chachi”	Daze, Jiangmen, Guangdong, China	Methanol	54.075 ± 0.578	[17]
C. reticulate “Chachi”	Yamen, Jiangmen, Guangdong, China	Methanol	88.087 ± 1.062	[17]
C. reticulate “Chachi”	Shuangshui, Jiangmen, Guangdong, China	Methanol	51.921 ± 0.768	[17]
C. reticulate “Chachi”	Siqian Town, Jiangmen, Guangdong, China	Methanol	50.137 ± 0.301	[17]
C. reticulate “Chachi”	Gujing, Jiangmen, Guangdong, China	50% Method	37	[18]
C. reticulate “Chachi”	Meijiang, Jiangmen, Guangdong, China	50% Method	42	[18]
C. reticulate “Chachi”	Wengyuan,Shaoguan, Guangdong, China	50% Method	79	[18]
C. reticulate “Chachi”	Xiaogan, Jiangmen, Guangdong, China	50% Method	30	[18]
C. reticulate “Chachi”	Shuangshui, Jiangmen, Guangdong, China	50% Method	53	[18]
C. reticulate “Chachi”	Wengyuan, Shaoguan, Guangdong, China	50% Method	66	[18]
C. reticulate “Chachi”	Shuangshui, Jiangmen, Guangdong, China	50% Method	46	[18]
C. Sinensis (linn.) Osbeck	North of Iran	Petrolatum ether first, followed by methanol	5.2-6.2	[19]
C. Sinensis	North of Iran		5.2-6.1	[19]
C. reticulate Blanco	North of Iran		4.5-6.1	[19]
C. unshiu Marc	North of Iran		1.3-5.8	[19]
C. unshiu Marc	Neretva Valley, Croatia	1.2M HCI in 80% methanol/water	0.42	[20]
C. reticulate Blanco	Neretva Valley, Croatia		0.47	[20]
lemon juice	Adana, Turkey		*∗*113.6 ± 11.0/litter	[21]
Pulp removing	Adana, Turkey		*∗*81.5 ± 12.7mg/litter	[21]
Pasteurization	Adana, Turkey		*∗*117.4 ± 32.5mg/litter	[21]
lemon	Spain		*∗*536 ± 234 mg/ml	[22]
Grapefruit	Spain		*∗*33 ± 3 mg/ml	[22]

^*∗*^Content in juice.

**Table 2:** Benefits of hesperidin on cutaneous functions.

Animal Models/Human Diseases/Cells	Treatment	Benefits	Mechanisms	Ref.
*Epidermal Permeability Barrier Function*				
Young mice	Topical applications of 2% hesperidin twice-daily for 6 days.	↑Acute barrier recovery	↑ Proliferation; ↑ Filaggrin expression; ↑ Lamellar body secretion.	[31]
Aged mice	Topical applications of 2% hesperidin twice-daily for 9 days.	↑ Acute barrier recovery; ↓ Skin surface pH;	↑ Differentiation; ↑ Lipid production; ↑ NHE1 and sPLA2 expression; ↑ Lamellar body formation and secretion	[32]
Glucocorticoid-treated mice	One hour after each topical application of 0.05% clobetasol propionate, 2% hesperidin was applied. Treatments were twice-daily for 9 days	↑ Acute barrier recovery; ↓ Skin surface pH;	↑ Proliferation; ↑ Filaggrin expression; ↑ Lipid processing; ↑ Antioxidation; ↑ Lamellar body formation and secretion; ↑ *β*-glucocerebrosidase activity.	[33]
*Protecting UV Irradiation*				
Keratinocytes	Keratinocytes first treated with 50 *μ*M hesperidin for 1 hr, followed by UVB irradiation (30 mJ/cm^2^)	↓ DNA damage ↓ lipid peroxidation ↓ Protein carbonylation ↓ Apoptotic index	↓ Absorb UVB ↓ Reactive oxygen species ↓ Bcl-2 expression ↓ BAX expression	[34]
	Keratinocytes were treated with hesperidin (220*μ*g/ml) for 24 hr, followed by UVA irradiation (10 J/cm^2^). Cells were incubated with hesperidin for additional 6 or 24 hr	↑ Cell viability ↓ MDA content ↓ TNF-*α*, IL-1*β* and IL-6 mRNA Levels	↑ SOD activity	[35]
	Keratinocytes were treated with hesperidin (50, 100, 200, 400, 600, 1 000mg/L) for 24 hr, followed by UVB irradiation (15 mJ/cm^2^).	↓ CXCR2 expression	Not determined	[36]
Dermal fibroblasts	Fibroblasts irradiated with UVA at dose 10 J/cm^2^ and treated with hesperetin containing extract at various concentration for 72 hr	↓ Matrix metalloproteinase expression ↓ *β*‐galactosidase expression ↑ Collagen biosynthesis	Not determined	[37]
	Pretreated fibroblasts with 3 and 30 *µM *hesperetin glucuronide, followed by UVA irradiation at 500 kJm^−2^	↓ Necrotic cell death	Not determined	[38]
Mice	Mice treated topically with hesperidin (3 mg/ml) daily for 10 days, 30 min after each application of hesperidin, mice were irradiated with 180 mJ/cm^2^ UVB.	↓ Skin erythema & edema ↓ Epidermal proliferation ↓ Lipid peroxidation ↓ Inflammation ↓ DNA damage	↑ Catalase and superoxide dismutase activity	[39]
	Mice were treated topically 1% hesperidin methyl chalcone before and after one irradiation with 4.14 mJ/cm^2^ UVB	↓ Cytokine expression ↓ Lipid peroxidation	↑ Nuclear factor erythroid 2-related factor 2; ↑ Glutathione peroxidase-1, glutathione reductase & heme oxygenase-1	[40]
	Hesperidin methyl chalcone at the dose of 300 mg/kg was intraperitoneally given 1 hr before and 7 hr after, irradiation with 4.14 mJ/cm^2^ UVB	↓ Skin edema, neutrophil recruitment and matrix metalloproteinase-9 activity; ↓ Cytokine expression; ↓ Myeloperoxidase activity; ↓ Lipid peroxidation	↑ Glutathione levels and catalase activity.	[41]
	Orally administered 0.1 mL of water containing 100 mg/kg body weight hesperidin daily, while mice were irradiated 3 times at 48 h intervals per week for 12 weeks. Does of UVB were increased 60 mJ/cm^2^ per exposure at week 1 to 90 mJ/cm^2^ at week 7.	↓ Transepidermal water loss; ↓ matrix metalloproteinase-9 expression & activity; ↓ Cytokine expression ↓ wrinkle formation ↓ Epidermal proliferation	↓ Phosphorylation of mitogen activated protein kinase & extracellular signal-regulated kinases	[42]
	Single UVB irradiation at dose of 180 mJ/cm^2^	↓ Cyclobutane pyrimidine dimers; ↑ p53 expression	Not determined	[43]
Guinea pigs	The dorsal skin was exposed to UVB 3 times a week (every other day) for 2 consecutive weeks. The total energy dose of UVB was 1 J/cm^2^ per exposure. One week later, 1% hesperetin was topically applied daily to the hyperpigmented areas (2 mg/cm^2^) for 4 successive weeks.	↓ Transepidermal water loss;	Not determined	[44]
*Pigmentation *				
B16 mouse melanoma cells	Cells incubated with 20 *μ*g/mL of *Citrus* extracts or 3-50 *μ*M of hesperetin for 48 hr.	↑ Melanin content; ↑ Tyrosinase protein; ↑ Tyrosinase activity.	↑ Melanogenesis-related proteins; ↑ *β*-Catenin expression; ↑ Phosphorylated glycogen synthase kinase-3*β*	[45, 46]
	Cells incubated with hesperidin (32.25mg/mL) for 3 days	Minimum inhibition of melanogenesis	Not determined	[47, 48]
	Cells were treated with hesperidin (50, 100, 200, 400, 600, 1000mg/L) for 24 hr, followed by UVB irradiation (15 mJ/cm^2^).	↓ Tyrosinase activity; ↓ Melanin content;	Not determined	[36]
	Cells incubated with 5-20 *μ*M hesperidin for 3 days	↑ Melanin content;	Not determined	[49]
	Cells incubated with 50 *μ*M hesperidin for 3 days	↓ Melanin content; ↓ Tyrosinase protein; ↓ Tyrosinase-related protein 1,2	↓ Melanogenesis-related proteins; ↑ p-Erk1/2; ↑ Proteasome activity	[50]
	Cells incubated with *Citrus* extracts (12.5, 25.0, and 50.0 *µ*g/mL) for 3 days	↓ Melanin content; ↓ Tyrosinase protein & activity; ↓ Tyrosinase-related protein 1,2	↓ Microphthalmia-associated transcription factor (MITF) proteins	[51]
Human epidermal melanocytes	Cells incubated with 3-50 *μ*M of hesperetin for 48 hr.	↑ Melanin content; ↑ Tyrosinase activity	Not determined	[46]
	Cells incubated with 50 *μ*M hesperidin for 3 days	↓ Melanin content; ↓ Tyrosinase activity	Not determined	[50]
	Cells incubated with 0.4mg/ml *Citrus* extracts for 3 days	↓ Tyrosinase activity	Not determined	[52]
Enzymatic assay of mushroom tyrosinase		↓ Tyrosinase activity	Not determined	[49]
		1mg/ml of *Citrus* extracts induced over 40% inhibition of tyrosinase activity	Not determined	[52]
		1.75 mg/ml of calamondin peel extract,containing hesperidin, induced 90% inhibition of tyrosinase activity	Not determined	[53]
Guinea pigs	The dorsal skin was exposed to UVB 3 times a week (every other day) for 2 consecutive weeks. The total energy dose of UVB was 1 J/cm^2^ per exposure. One week later, 1% hesperetin was topically applied daily to the hyperpigmented areas (2 mg/cm^2^) for 4 successive weeks.	↓ Pigmentation	Not determined	[44]
Reconstructed human epidermis	The epidermis was treated topically with 0.2% hesperidin for 14 days	↓ Pigmentation	Not determined	[49]
Humans	Topical applications of cream containing 0.4 mg/ml *Citrus* extracts for 56 days	>8% increase in skin brightening	Not determined	[52]
*Cutaneous Wound Healing*				
Dermal fibroblast	Fibroblasts were incubated with mixture containing 0·05 mg/ml hesperidin for 24 or 96 hr.	↑ Wound closure.	↑ Collagen synthesis	[54]
Diabetic rats	After wound, rats were given oral hesperidin (25-100 mg/kg body weight) for 21 days.	↓ Wound closure.	↑ VEGF-c, Ang-1/Tie-2, TGF-*β* and Smad-2/3 mRNA expression; ↑ SOD and GSH levels; ↓ MDA and NO levels;	[55]
	After wound, rats were given oral hesperidin (10-80 mg/kg body weight) for 20 days.	↑ Wound closure.	↑ VEGFR1 and VEGFR2 levels; ↓ TNF*α*, IL-6; ↑ SOD and GSH levels; ↓ MDA levels;	[56]
Humans with venous ulcers	Fifteen patients were treated orally with diosmin/hesperidin (450/50 mg, twice daily) for 90 days. Another 15 patients treated with pycnogenol (50 mg orally, 3 times daily) served as controls	No differences in wound healing time between two groups	Not determined	[57]
	Fifty-three patients received Daflon 500 mg, and 52 received placebo for 2 months	↓ Wound healing time; ↓ Hospitalization duration	Not determined	[58]
*γ* irradiated mice	Mice were given oral hesperidin (100mg/kg body weight) once 1 hr before *γ* irradiation. Wound was made prior to irradiation.	↑ Wound contraction; ↓ Wound healing time.	↑ NO; ↑ DNA synthesis; ↑ Collagen; ↑ Hexosamine; ↑ Densities of bold vessels and fibroblasts	[59]
	Mice given 1, 2, 5 or 10 % of hesperidin ointment topically covering the whole excision wounds, twice daily after exposure to 6 Gy ${\hat{I}}^{3}$-radiation until complete healing of wounds.	↑ Wound contraction; ↓ Wound healing time.	Anti-oxidative stress	[60]
*Inflammation*				
Mouse RAW 264.7 cell line	Incubated with hesperidin (5-250 *μ*g/ml)	↓ Lipopolysaccharide-induced nitric oxide production	Not determined	[30]
	Incubated with hesperidin or hesperetin (40-100 *μ*M) for 30 min, followed by stimulation with 1 *μ*g/mL of Lipopolysaccharide	↓ Antioxidative stress; ↓ PGE2; ↓ COX‐2 expression; ↓ Nitric oxide production	↓ NF‐*κ*B activation; ↓ JNK1/2 and p38 phosphorylation; ↓ I*κ*B*α*; ↓ iNOS mRNA; ↓ Antioxidative stress;	[61]
Keratinocytes	Keratinocytes treated with HES (20 *µ*g/mL) for 2 hr, followed by incubation with H_2_O_2_ for 48 hr	↓ IL-8 protein & mRNA; ↓ TNF‐*α* protein & mRNA; ↓ COX‐2 expression	↓ NF‐*κ*B activation, phosphorylated I*κ*B*α* and phosphorylated p38 MAPK	[62]
	Cells were incubated with both heat-killed Propionibacterium acnes and 5-50 *µ*g/mL of hesperidin for 24 hr.	↓ IL-8 protein & mRNA; ↓ TNF‐*α* protein & mRNA;	Not determined	[63]
Human skin explants	Human skin explants were pre-incubated with hesperidin methyl chalcone (0.2 mg/ml) and then stimulated with SP for 24 hours.	↓ Proportion of dilated vessels; ↓ Total vessel area; ↓ IL-8 production.	Not determined	[64]
Rats	Thirty min prior to carrageenan or dextran injection, hesperidin (50 or 100 mg/kg body weight) was subcutaneous injected	↓ Edema	Not determined	[65]
Mice	Intraperitoneal injection of hesperidin (75 mg/kg), following by subcutaneous injection of carrageenan	↓ Edema	Not determined	[66]
Guinea pigs	Hesperidin (40mg/kg) was orally given 1 hr prior to injection of carrageenan.	↓ Edema	Not determined	[67]
*Skin Cancers*				
A431 human skin carcinoma cells	Incubation of cells with hesperetin (10,100,500 *μ*M) for 24 hr	↑ DNA fragmentation; ↑ Apoptotic proteins (p21 and Bax); ↓ levels of cyclin A2, B1, D1, D3 and E1	↑ ERK, JNK, p38, ROS	[68]
	Cells treated with hesperidin (10, 25 and 50 *μ*M) for various times	↓ Cell viability; ↓ Levels of cyclin D, CDK2 and thymidylate synthase; ↑ Apoptosis	↑ ROS ↓ ATP content	[69]
Mice	Subcutaneous injection of 125 *μ*l of 1% hesperidin solution daily 1 week prior to tumor induction.	↓ Incidence of tumor and number of tumor per mouse	Not determined	[70]

Note: SOD: superoxide dismutase; GSH: reduced glutathione: NO: nitric oxide (NO); PGE2: prostaglandin E2; VEGF: vascular endothelial growth factor; MPO: myeloperoxidase; MDA: malondialdehyde*; *COX2: *cyclooxygenase-2, COX-2; SP: substance P; DMBA: 7,12-dimethylbenz[a]anthracene; TPA: 12-O-tetradecanoyl-13-phorbol acetate; ROS*: reactive oxygen species; ERK: extracellular signal-regulated kinase; JNK: c-Jun NH2-terminal kinase; CDK2: cyclin-dependent kinase 2.

## Abbreviations

VEGF: Vascular endothelia growth factor
SOD: Superoxide dismutase
GSH: Reduced glutathione
MDA: Malondialdehyde
TGF-*β*: Transform growth factor *β*
MAPK: Mitogen-activated protein kinases.
